# New Insights Into the Cancer–Microbiome–Immune Axis: Decrypting a Decade of Discoveries

**DOI:** 10.3389/fimmu.2021.622064

**Published:** 2021-02-23

**Authors:** Tejeshwar Jain, Prateek Sharma, Abhi C. Are, Selwyn M. Vickers, Vikas Dudeja

**Affiliations:** Department of Surgery, University of Alabama at Birmingham, Birmingham, AL, United States

**Keywords:** gut microbiome, cancer, tumor microenvironment, microbiome-immune crosstalk, cancer immunotherapy, immune system

## Abstract

The past decade has witnessed groundbreaking advances in the field of microbiome research. An area where immense implications of the microbiome have been demonstrated is tumor biology. The microbiome affects tumor initiation and progression through direct effects on the tumor cells and indirectly through manipulation of the immune system. It can also determine response to cancer therapies and predict disease progression and survival. Modulation of the microbiome can be harnessed to potentiate the efficacy of immunotherapies and decrease their toxicity. In this review, we comprehensively dissect recent evidence regarding the interaction of the microbiome and anti-tumor immune machinery and outline the critical questions which need to be addressed as we further explore this dynamic colloquy.

## Introduction

It is an oft-quoted fact that the human body has more bacteria as compared to human cells (1). Indeed, the human body is a giant collaboration of symbiotically thriving microbes and hosts cells, constantly interacting with one another. Interestingly, within the ecosystem of the human body, various microbial species colonize distinct niches, but their abundance and functions are liable to fluctuations under the influence of numerous exogenous and endogenous cues. Investigations into these alterations have uncovered the enormous impact of the microbiome on human health and disease (2, 3). Although the scientific community has not been able to characterize the ‘healthy’ microbiome or eubiosis, distinct ‘dysbiotic’ microbial signatures associated with disease states are being increasingly recognized using high throughput sequencing techniques. This has led to delineation of the role of the commensal microbiome in modulating disease progression spanning various pathologies and systems, including cancer.

Genetic mutations have been considered as the main drivers of tumor initiation, with contributions from secondary risk factors like diet, age, lifestyle factors, microbes *etc*. However, we now know that the microbiome can regulate the effects of tumor-driver mutations as well (4, 5). Many of the important life-style risk factors for cancer like obesity (6), smoking (7), diet (8) and alcohol (9) can cause perturbations in the microbial composition as well. Emerging evidence suggests that host microbial signatures may be able to predict some incipient cancers (10), modulate response to and toxicity of cancer immunotherapy (11–13) and even correlate with survival in specific cancers (14, 15). Extensive exploration of the role of the microbiome in cancer evolution has demonstrated that while the microbiome can affect cancer cells themselves, it can also modulate the cancer immunosurveillance. In this review, we comprehensively describe the microbiome–immune interaction in the homeostatic state as well in cancers. We discuss the various direct and indirect mechanisms by which this immunomodulation occurs and their implications for tumor growth and therapy. Finally, we outline the future directions microbiome research might head towards and the challenges that need to be overcome for deconvoluting this complex crosstalk.

## The Microbiome: A Diverse Consortium With Diverse Health Implications

The human microbiome is defined as the collection of all the microorganisms living in association with the human body including the skin, mammary glands, placenta, seminal fluid, uterus, ovarian follicles, lung, saliva, oral mucosa,conjunctiva,biliary tract, and gastrointestinal tract. These communities include eukaryotes, archaea, bacteria, and viruses. Although the number of microorganisms that we harbor is still an unanswered question, what is known with conviction is that they are extremely diverse. It is estimated that approximately 500–1,000 bacterial species exist in the human body at any one time, and there could be several more unique genotypes (subspecies) within them. Each bacterial species has thousands of genes within its genome and that itself makes just the collective bacterial genome several hundred times more than the commonly estimated 20,000 human genes. Between individuals, the diversity among the microbiome is immense compared to genomic variation: individual humans are about 99.9% identical to one another in terms of their host genome (16), but can be 80–90% different from one another in terms of the microbiome of their hand (17) or gut (18). This gives an opportunity to target the more flexible gut microbiome than approaches that target the relatively constant host genome. The microbiome is in a constant state of flux, and it fluctuates with nutrition, age, geography, use of antibiotics/probiotics and other environmental influences. Importantly, we still do not know clearly how this variation in microbiota influences wellness or onset/progression of diseases. However, recent years have seen an increase in our understanding of the role of gut microbiome not only in health but also in the pathogenesis of various disorders like inflammatory bowel disease, ischemic stroke (19), NASH (20), hepatic fibrosis (21) and obesity (22, 23). As such, our understanding of the role of gut microbiome in the pathogenesis of various diseases is continually evolving, with newer data implicating its role in an expanding number of conditions. The association of microbiota and cancer has been known since the early 19^th^ century with the discovery of chicken sarcoma virus, capable of transmitting sarcoma to healthy chickens (24). This was followed by demonstration of the tumorigenic potential of agents like Epstein Barr virus, mammary tumor virus and *H. pylori*. With improvements in next generation sequencing technologies in the 21^st^ century, plethora of evidence has emerged regarding the role of microorganisms in carcinogenesis. A summary of the studies highlighting this relationship can be found in **Table 1**.

**Table 1:** Role of the microbiome in cancer progression.

Cancer Type	Study	Model	Microbes indicated	Mechanism of Microbial modulation	Key finding
**Glioma**	Alessandro et al. (25)	Intracranial injection of syngeneic glioma cell line	Gut commensals	–	Antibiotic administration enhanced intracranial glioma growth and reduced cytotoxic NK cell subset.
**Lung cancer**	Jin et al. (26)	Genetically engineered KP (Kras*^LSL-G12D^*; p53*^flox/flox^*) model of lung adenocarcinoma	Lung commensals	Commensal bacteria produce an immunosuppressive TME by induction of IL-17 producing *γδ* T cells	Gut microbiome depletion led to anti-tumor response through IFN-*γ* up regulation in *γδ* T cells
**Melanoma**	Li et al. (27)	Subcutaneous injection in WT, *Rnf5*^−/−^ and Germ-free mice	–	Altered UPR signaling as seen in *Rnf5*^−/−^ mice, coincides with altered gut microbiota composition and anti-tumor immunity to control melanoma growth	Transfer of 11 bacterial strains, including *B. rodentium*, enriched in *Rnf5*^−/−^ mice, establishes anti-tumor immunity and restricts melanoma growth in WT germ free mice.
	Sethi et al. (28)	Subcutaneous injection of melanoma cells derived from Tyr-CreER; Braf; Ptenfl/fl mice; intrasplenic injection of B16-F10 melanoma cells	–	–	Antibiotic cocktail decreased primary tumor growth and hepatic metastasis burden
**Melanoma Lung metastases**	Cheng et al. (29)	Intravenous injection of B16 melanoma cells	–	–	Antibiotic administration impaired functional *γδ* T cell response and enhanced lung metastases
	Noci et al. (30)	Intravenous injection of B16 melanoma cells	Lung commensals	Commensal induced Foxp3^+^ T_regs_ led to enhanced metastatic growth	Aerosolized vancomycin/neomycin downregulated Foxp3^+^ T_regs_ and increased T cell and NKT infiltration with decreased metastatic growth. Aerosolized *Lactobacillus rhamnosus* recapitulated the antitumor effects of antibiotics.
**Breast cancer**	Rao et al. (31)	*Apc^Min/+.^* mice gavaged with *Helicobacter* *hepaticus*	*Helicobacter* *hepaticus*	–	*H hepaticus* infection induced mammary (and intestinal) tumor
	Rutkowski et al. (32)	Flank injection of syngeneic cancer cell lines in C57BL/6J mice	Gut commensals	TLR 5 dependent commensal bacteria drive malignant progression by increasing systemic IL-6 and MDSC recruitment	Depletion of commensal bacteria abrogates TLR 5 dependent difference in tumor growth.
	Parhi et al. (33)	Orthotopic injection (mammary fat pad) of syngenic cell lines in BalB/c and C57BL/6J mice	*Fusobacterium nucleatum*	*F. nucleatum* colonizes breast cancer tissue through its Fap 2 lectin and suppresses T cell infiltration and promotes tumor growth	Antibiotic administration abrogated *F. nucleatum* induced tumor progression
	Kovacs et al. (34)	Orthotopic injection (mammary fat pad) of 4T1 cell line in BalB/c	Cadaverine- microbial metabolite	–	Cadaverine treatment decreases tumor growth, EMT and invasiveness through trace amino acid receptors in murine model. Early breast cancer patients had reduced expression cadaverine production genes in stool.
**Liver cancer**	Ma et al. (35)	Spontaneous liver metastatic from subcutaneous sites, Myc transgenic mice, intrasplenic, and tail vein injection of cancer cell lines	*Clostridium* sp.	Antibiotic administration induced accumulation of cytotoxic NK T cells by increasing primary to secondary bile acid fraction.	Antibiotics significantly decreased liver tumor burden
	Fox et al. (36)	Aflatoxin B1-induced liver cancer in C3H/HeN mice	*H hepaticus*		*H hepaticus* increased the hepatocellular carcinoma tumor burden in aflatoxin-treated mice
	Dapito et al. (37)	Diethylnitrosamine and carbon tetrachloride administration to mice	*-*	HCC-promoting effects of intestinal microbiota needed activation of factors downstream of TLR4	Antibiotics-treated mice and GF mice had decreased HCC burden Lipopolysaccharide treatment exacerbated tumors
**Gastric cancer**	Wang et al. (38) Fox et al. (39)	Insulin-gastrin (INS-GAS) transgenic mice	*Helicobacter felis* *Helicobacter pylori*	–	Helicobacter infection exacerbated tumor progression in mice
	Wong et al. (40)	Randomized controlled trial (1630 carriers of H pylori infection intervened with *H. pylori* eradication treatment or placebo)	*Helicobacter pylori*	–	In the subgroup of patients who had no premalignant lesion on induction of study, no patient receiving *H. pylori* eradication treatment developed gastric cancer compared to 6 controls who did (p = 0.02)
**Low-grade gastric** **mucosa-associated** **lymphoid tissue (MALT)** **lymphoma**	Rogero et al. (41)	Prospective Cohort (26 patients with *H. pylori* infection and evidence of low-grade gastric MALT lymphoma)	*Helicobacter pylori*	–	Cancer regressed in 15 of 25 patients who showed evidence of complete eradication of H pylori (60%; 95% CI, 39 to 79%)
**Colorectal cancer**	Kado et al. (42)	Spontaneous adenocarcinoma in TCRb−/− p53−/− mouse	*-*	–	70% of conventional but no GF mice developed colon adenocarcinoma
	Grivennikov et al. (43)	Cdx2-Cre; Apcf/wt (CPC-APC) mice	*-*	Antibiotics mediated anti-tumor effects were abrogated in IL23r−/− mice	Antibiotics decreased colorectal cancer burden. Bacterial presence in colonic tumors from mice and humans
	Arthur et al. (44)	AOM to Il10−/−mice	*Escherichia coli*	Inflammation was necessary for pks. bacteria-induced carcinogenesis	Monocolonization with pks. *Escherichia coli* induced adenocarcinomas
	Dejea et al. (45)	AOM-treated SPF mice	Enterotoxigenic *B. Fragilis* *Pks E coli*	IL17 depletion abrogated bacteria induced tumorigenesis.	Co-colonization of mice with biofilm bacteria, E Enterotoxigenic *B. Fragilis* and pks. *E coli* significantly increased tumorigenesis
	Abed J et al., Gur C et al., Kaplan CW et al., Kostic et al., Park et al., Hamada et al. (46–51)	Apc (Min/+) Orthotopic rectal injection Human colorectal cancer tissue	*Fusobacterium species*	Multiple immuno-suppressive adaptations, *vis-à-vis* inhibiting T-cell activity and NK cell cytotoxicity, increasing myeloid-derived suppressor cells and tumor-associated macrophages as well as suppressing TILs in MSI-high subtype of colorectal cancer	*F. nucleatum* homes to colorectal cancer tissue and causes cancer progression by inducing immunosuppression.
	Tanoue et al. (52)	MC-38 subcutaneous model	Mix of 11 human commensal bacterial strains	Induction of IFN*γ*^+^ CD8 T cells both locally and systemically by the 11-mix strains	Inoculation of this 11-mix strain into GF mice significantly decreased tumor growth and enhanced immunotherapy response
**Pancreatic cancer**	Pushalkar et al. (53)	Ptf1aCre;LSL-KrasG12D (KC) mice; wild-type mice implanted orthotopically with KPC cell lines	*Bifidobacterium pseudolongum*	Microbial sensing through TLR2 and TLR5 induced intratumoral immunosuppression	Pancreatic tumors harbored bacteria GF status or antibiotics treatment decreased pancreatic cancer burden.
	Sethi et al. (28)	Subcutaneous implantation and intrasplenic injection of KPC cells	*-*	Gut commensals promoted tumor growth in an IL-17 dependent manner	Antibiotic treatment decreased primary tumor growth and hepatic metastasis burden
	Thomas et al. (54)	KrasG12D/Ptenlox/. mice; Nod- SCID mice subcutaneously implanted with BxPC3 and L3.6pl human cell lines	*-*	Increased immune infiltration	Antibiotiv treatment decreased pancreatic cancer burden
	Aykut et al. (55)	Ptf1aCre;LSL-KrasG12D (KC) mice; wild-type mice implanted orthotopically with KPC cell lines.	*Malassezia sp*	Ligation the Mannonse-binding lectin and upregulation C3 complement cascade by fungal sp.	Pancreatic tumors harbored fungal sp. Antifungal treatment decreased pancreatic cancer burden
**Various**	Sacksteder et al. (56) Reddy et al. (57) Laqueur et al. (58)	Natural incidence of tumors in inbred GF rats observed over generations. Carcinogen exposure to rats (3,20-dimethyl-4-Aminobiphenyl; Methylazoxymethanol-b-D glucosiduronic Acid)	–	–	Decreased natural incidence of diverse tumors in GF rats compared to historical data. Also, decreased tumor development in GF rats compared to conventional rats on exposure to carcinogens.

## Role of the Commensal Microbiome in Normal Immune Maturation

There exists a co-evolved relationship between microbiota and both the innate and adaptive immune systems. Studies on germ-free animals (GF) done in the 1950s and 1960s provided the earliest evidence connecting the exposure to various microbes and the development of a robust immune system. GF mice harbor a multitude of defects in their immune system and are more prone to infections. They not only lack mucosal immunity (59), but also demonstrate other immune deficits including smaller Peyer’s patches, decreased mesenteric lymph nodes, lack of lymphoid follicles in the lamina propria (LP), over-activation of anti-inflammatory T helper (Th) type 2 cytokines, and decreased expression of pattern recognition receptors (PRRs), such as Toll-like receptors (TLRs) (60, 61). Interestingly, when these GF mice are transplanted with microbiota from standard pathogen free (SPF) mice (conventionalization), these defects are overcome, and immune maturation occurs. In terms of innate immune system education, gut microbiota have been shown to enhance myelopoiesis and maturation of myeloid cells (62, 63), induce functional innate lymphoid cells (ILCs) (64) and influence maturation and functions of Kupffer cells in liver sinusoids (65, 66). Thus, it appears that microbes are involved in the maturation of both innate and adaptive immune systems.

Research evaluating the interaction of TLRs (67) and adaptive mucosal immunity of the gut mucosa has shed further light on microbiome based immune-education. Gut lamina propria is the richest source of lymphocytes in the human body, where different arms of the adaptive immune system (Tregs, Th1, Th2, Th17) exist in a delicate balance. Beura and colleagues (68) made a seminal observation that while wild mice and adult humans have highly differentiated memory CD8+ T cell compartment in blood, these mature effector cells are absent in laboratory mice as well as human neonates. Interestingly, cohousing feral mice with laboratory mice led to horizontal transfer of microbiota, and simultaneously matured the immune system and led to increased resistance to influenza virus in laboratory mice. Th_9_ cells are a distinct subset of CD4^+^ T cells which differentiate under the influence of IL-4 and TGF-ß and secrete IL-9 (69, 70) and play an important role in anti-tumor immunity (71, 72). The gut microbiome can also influence Th_9_ development, as GF mice and antibiotics treated SPF mice have decreased colonic expression of IL-4 and TGF-ß, thus reducing IL-9 secreting T cell population (73).

In fact, the literature is replete with data on certain microbes promoting differentiation of specific immune lineages. For instance, it has been observed that *Bacteroides fragilis* and *Clostridia* can suppress colitis by induction of Foxp3^+^ Tregs in the colon (74, 75). *Bacteroides fragilis* has also been shown to induce systemic immunological maturity of GF mice and increase the splenic CD4^+^ fraction to the level seen in SPF mice (76). Tanoue et al. have now identified specific commensal colonies which have the ability to induce IFN*γ*^+^ CD8 T cells in GF mice, both locally (in the colonic lamina propria) as well as systemically. Using multiple mice models, they isolated a mixture of 11 microbial strains (seven *Bacteroides* species—*Parabacteroides distasonis, Parabacteroides gordonii, Alistipes senegalensis, Parabacteroides johnsonii, Paraprevotella xylaniphila, Bacteroides dorei, Bacteroides uniformis JCM 5828*; four non-Bacteroides—*Eubacterium limosum, Ruminococcaceae bacterium cv2, Fusobacterium ulcerans, Phascolarctobacterium faecium*), which are represented sparsely in the human gut and can incite this CD8 T cell induction, with a relative enrichment for the TCR V*β*6^+^ and V*β*8^+^ subsets (52). It was also recently shown that the site of microbial exposure can determine the B cell repertoire in terms of both specificity and diversity in GF mice (77). Expectedly, transient mucosal exposure to microbial species induces an IgA predominant repertoire, while systemic exposure induces an IgM–IgG predominant repertoire in GF mice. Intriguingly, heavy chain sequences from the IgA class-switched B cells upon mucosal exposure show many similarities with the IgA class-switched B cells from GF mice, while systemic exposure induced IgG-switched B cells do not share many similarities with their GF counterparts. Moreover, IgA repertoire does not expand after dose escalation of the mucosal exposure but the IgG repertoire significantly diversified on escalating systemic doses (77). These findings indicate a possible evolutionary adaptation to generate a restricted mucosal IgA repertoire to tolerate gut commensals while maintaining a flexible and diverse IgG repertoire to combat invasive infections. Interestingly, genetically identical mice purchased from different laboratory vendors can have unique baseline and stimulated immune response, which again is guided by their unique gut microbiome composition. For instance, mice obtained from Taconic, when compared to those from Jackson, have higher baseline level of Th-17 cells, and this difference is driven by presence of *Candidatus arthomitus*, a segmented filamentous bacterium, in the gut microbiome of the Taconic mice (78, 79). This raises a very intriguing possibility that the studies exploring immune response may have to evaluate the result in context of gut microbial composition and other determinants of immune response.

It appears that microbial metabolites may help explain this role of gut microbes driving immune maturation. For instance, Bachem et al. have demonstrated that microbiota-produced butyrate is essential for survival and memory responses of activated CD8 T cells, where it can uncouple TCA from glycolysis to increase oxidative phosphorylation and glutamine and fatty acid metabolism. This results in decreased proliferation, increased responsiveness to IL-15, and upregulated FoxO1 expression, thus resulting in enhanced memory response (80). Butyrate and propionate (metabolites produced by fiber-fermenting gut microbes) can induce extra-thymic Tregs by virtue of their histone deacetylation (HDAC) inhibitory activity (81). Butyrate production from microbiota has also been linked to upregulated Foxp3 expression and IL-9 repression in Th9 cells during lung inflammation (82). Intuitively, the microbiome-immune system interaction is bidirectional in nature, with gut microbiome helping with immune maturation and immune system helping sculpt gut microbiome composition. For instance, depletion of Foxp3^+^ T_regs_ can lead to long term enrichment of *Firmicutes* as well as transient changes in *Prevotella*, *Akkermansia*, and *Oscillospira* (83).

Excitingly, epidemiological human studies also provide support to the important role of the gut microbiome in shaping the immune system. For instance, there exists a stark difference in resident gut flora in neonates delivered *via* cesarean, with colonization by mostly skin commensals (*Staphylococcus, Corynebacterium*), when compared to the neonates born by normal vaginal delivery who have the normal flora of the adult vagina (*Lactobacilli, Prevotella* spp) dominating in their gut microbiome (84). While the differences observed in the gut microbiome composition due to mode of delivery narrows down with advancing age, one can still detect differences at 7 years of life with a higher proportion of *clostridium* sp. in vaginally delivered neonates (85). Interestingly, a frequent argument is raised that administration of intrapartum antibiotics during caesarian delivery contributes to the difference in gut microbiome composition. However, a recent study by Reyman et al. (86) has shown that gut microbiome profoundly differs at one week of life between the two modes of delivery independent of administration of intrapartum antibiotics. Additionally, the microbiome composition at this early time point is associated with the number of respiratory infections. A child will suffer during the first year of life with the taxa strongly associated with infections being enriched in cesarian delivered neonates. Studies have reported cesarean delivery to be associated with increased risk of developing autoimmune diseases later in life, such as diabetes, asthma, inflammatory bowel diseases, food allergies, and juvenile arthritis, when compared to vaginally delivered neonates (87–90). Similarly, exposure to antibiotics early in life can have a long lasting effect on gut microbiome composition and has been associated with increased risk of development of IBD (91), obesity (92), and cancer (93). Yet another evidence comes from the protective effects of breast milk during infancy. Exclusive breastfeeding provides a proliferative stimulus for *Bifidobacterium* in the neonatal gut, which is associated with protection against necrotizing enterocolitis (94) and decreases the risk of asthma later in life (95). However, these studies are merely associative and do not establish causality between gut microbiome differences in development of autoimmune diseases and need prudence in interpretation, as there might be other factors at play.

## The Immune System and Cancer

It was more than a century ago when Paul Ehrlich first hypothesized that the immune system suppressed the growth of carcinomas (96). Technological limitations precluded our ability to investigate this hypothesis until the last decade of 20^th^ century. Since then, tremendous work has been undertaken in tumor immunology, giving rise to the concept of cancer immunoediting (97). Cancer immunoediting comprises the interactions of cancer with the immune cells and is divided into three phases—‘elimination’, ‘equillibrium’, and ‘escape’. Constant surveillance by the adaptive immune cells and subsequent cytotoxicity through production of effectors like IFN-*γ* is responsible for elimination of the cancer cells. However, tumors develop mechanisms to evade this surveillance through tumor intrinsic pathways (98) as well as through interaction with the immune cells and other components of the tumor stroma in the tumor microenvironment (99). During tumor growth, certain immune cells in the tumor microenvironment can even become tumor-promoting under the influence of local cytokines and other metabolic products (99). Highlighting the importance of these interactions, numerous studies have identified molecular pathways which can be targeted to overcome this immunoresistance and constrain tumor growth (100). Promising new therapies aiming to potentiate the anti-tumor immune response, like immune checkpoint blockade inhibitors (101), CAR-T cells (102), CpG oligonucleotide (103) therapies have been developed and are being evaluated in clinical trials. Not only immunotherapies, a functional immune system is also important for the effects of other anti-tumor therapies like chemotherapy (104) and radiotherapy (105). In light of the intimate association of the microbiome with our immune system, there has been a recent interest in elucidating the significance of this interaction for cancer growth and therapy (106).

## Microbial Inflammation: Pro-Tumorigenic or Anti-Tumorigenic?

The role of inflammation in the pathogenesis of cancer is well established. Chronic inflammation cultivates a fertile bed for tumor initiation and growth, as evidenced by increased cancer incidence in multiple chronic inflammatory conditions like IBD, ulcerative colitis, pancreatitis, chronic atrophic gastritis, *etc* (107). Similarly, microbe-induced inflammation has traditionally been considered as pro-tumorigenic. Indeed, initial examples of cancers associated with microbes visibly encapsulated this infection-inflammation-cancer continuum, a case in point being *H. pylori* gastritis (108, 109) and *Schistosoma haematobium* infection (110, 111) leading to the development of gastric and bladder cancer respectively. Colon cancer is yet another example where microbes modulate the process of cancer development and progression. For instance, *Fusobacterium* species inside colorectal tumors can promote tumor growth by instigating multiple immuno-suppressive adaptations, *vis-à-vis* inhibiting T-cell activity and NK cell cytotoxicity (46–48), increasing myeloid-derived suppressor cells and tumor-associated macrophages (49, 50) as well as suppressing TILs in MSI-high subtype of colorectal cancer (51). Like colon, lungs are also present at the interface of the environment with the body and routinely have microbial colonization. Intriguingly, it has been demonstrated that the homeostatic immunoregulatory effects of local commensals can inadvertently cultivate pro-tumorigenic milieu in lungs. Normally, local flora in lungs is thought to contribute to induction of regulatory T cells to suppress excessive inflammatory responses against the continuous inflow of inhaled foreign antigens (112–114). Using models of metastatic melanoma, Noci et al. showed that commensal-induced Foxp3^+^ T_regs_ could lead to increased metastatic tumor growth in the lungs and modulation of the local pulmonary microbiota using aerosolized vancomycin or neomycin downregulated IL-10 producing Foxp3^+^ T_regs_ population, which led to increased T cell and NK T cell infiltration as well as reduced metastatic nodules (30).

Recent years have seen the discovery of the role of microbes in initiation and development of cancer at sites which till date were considered sterile. For instance, microbes have recently been shown to drive pancreatic cancer initiation and progression in pre-clinical models by creating an immunosuppressive tumor microenvironment through increased MDSC recruitment and suppression of Th_1_ immune response (53) as well as stimulating IL-17 secretion (28). These immunosuppressive effects also contribute to the lack of efficacy of immunotherapy in PDAC (covered in greater details later) (28, 53, 115). *Fusobacterium nucleatum*, an oral anaerobic commensal, has been shown to home preferentially to malignant mammary tissue as compared to normal adjacent tissue through binding of bacterial Fap2 to tumor Gal-GalNAc sites, and induce a pro-tumorigenic inflammation by reducing CD 4^+^ and CD8^+^ T cell infiltration, thus leading to increased tumor growth and metastasis (33). Similar effect was not shown by other anaerobic oral commensals like *P. gingivalis*, highlighting a specific affinity between pathobionts and tumors (33). In addition to affecting well-established tumors, the gut microbiome has been shown to drive pro-tumorigenic inflammation in precursor lesions as well. A recent study has found increased incidence of oral microbes including *F. nucleatum* in resected specimens of cystic lesions of human pancreas. These bacteria were significantly enriched in patients with high grade intraductal papillary mucinous neoplasms (IPMNs) and invasive cancer as compared to patients without IPMNs. Moreover, there was an increased intra-cystic IL-1*β* in the high grade IPMNs and invasive cancer groups which correlated positively with bacterial 16s DNA content, indicating that the bacterial presence in the cysts might be driving the increased levels of pro-tumorigenic cytokines like IL-1*β* even at a precursor lesion stage (116).

While overwhelming evidence points towards an oncogenic role of microbiome-driven inflammation, recent studies have demonstrated some very intriguing examples (although indirect) of potential anti-cancer roles of microbial inflammation. For instance, Riquelme et al. recently noticed that intra-tumoral microbial diversity correlated with survival of pancreatic ductal adenocarcinoma (PDAC) patients, with significant enrichment of Proteobacteria (*Pseudoxanthomonas*) and Actinobacteria (*Saccharopolyspora* and *Streptomyces*) in the long-term survivors. Moreover, the long-term survivors had an increased density of CD3^+^ and CD8^+^ T cells as well as Granzyme B^+^ cells in the TME (15). Furthermore, the pro- *vs* anti-cancer role may depend on the specific bacterial community. In contrast to the pro-tumorigenic inflammation induced by *Fusobacterium* in CRC, other bacterial species have been shown to be associated with anti-cancer inflammation. It has been demonstrated that specific bacterial species in human colon cancer biopsies correlate with prognostically favorable increase in T cell infiltration by virtue of increased expression of T cell recruiting chemokines by the cancer cells (*Lachnospiraceae* and *Ruminococcaceae*) and increased chemokine receptor expression on multiple T cell subsets (*Methylobacteriaceae*) (117). Preliminary findings from a preclinical glioma model suggest that treatment of tumor bearing mice with vancomycin plus gentamicin decreased gut microbial diversity, decreased infiltration of CD27^+^CD11b^+^ cytotoxic NK cells and led to increased tumor burden (25). Another specific interaction in seen is the form of molecular mimicry, where microbial epitopes can mimic tumor antigens, leading to enhanced anti-tumor immunity through generation of microbial epitope specific activated CD8^+^ T cells (discussed later) (118).

## Effects of the Microbiome on Specific Immunocytes During Cancer Progression

During cancer initiation and progression, manipulation of the innate and/or the adaptive immune system by microbes, can transform the relationship between cancer and immune system and modulate cancer immunosurveillance (**Table 2**). In fact, as described below, microbes have been shown to affect the response of all kinds of innate and adaptive immune effectors to cancer.

**Table 2:** Modulation of the immune tumor microenvironment by the microbiota.

Immune cells	Study	Cancer	Immunodulatory effect
INNATE IMMUNE SYSTEM			
**Dendritic cells**	Paulos et al. (119)	Melanoma	TLR4 signaling leading to activated dendritic cells and increased antigen presentation to adoptively transferred CD8 T cells post irradiation
	Uribe-Herranz et al. (120)	Melanoma, Lung, Cervical	Butyrate secreted by gut commensals inhibits antigen cross priming of adoptively transferred CD8 T cells by CD11c^+^ dendritic cells post radiation therapy
**NKT cells**	Ma et al. (35)	HCC	Gut commensals metabolize 1° bile acids into 2° bile acids, impairing NKT mediated immunosurveillance of hepatic tumors in a CXCL16 dependent manner
	Gur et al. (47)	Colon	Fap2 protein of *F. nucleatum* binds to TIGIT to inhibit NKT cell cytotoxicity
**Myeloid cells**	Li Rui et al. (121)	Colon	Cathepsin K (CTSK) secreted upon gut microbial dysbiosis induces M2 polarization in TME through TLR4 signaling
	Pushalkar et al. (53)	PDAC	TLR2 and TLR5 mediated M2 polarization of intra-tumoral macrophages to induce Th2 adaptive immune response
	Kostic et al. (49)	Colon	*F. nucleatum* recruits arginase-1 producing MDSCs and polarizes macrophages to M2 phenotype in TME
	Rutkowski et al. (32)	Sarcoma	TLR5 dependent IL-6 upregulation systemically induces MDSCs infiltration into tumors, leading to galectin-1 secretion by *γδ* T cells suppressing anti-tumor immunity
	Iida et al. (122)	Colon, Melanoma	Abx-mediated gut microbial depletion decreases TNFα secreting myeloid cells in the TME, thereby reducing the efficacy of anti-IL10R/CpG ODN combination immunotherapy
**Complement system**	Aykut et al. (55)	Pancreatic cancer	*Malassezia* sp can promote pancreatic oncogenesis by ligating Mannose-binding lectin in the TME and activating C3 complement cascade
ADAPTIVE IMMUNE SYSTEM			
**CD8^+^ T cells**	Tanoue et al. (52)	colon	A mix of 11 specific gut commensals elicits an enhanced IFN-*γ*^+^ CD8T cells intratumorally, relatively enriched for the V*β*13^+^ subset of TCR, along with improved immunotherapy efficacy
	Sethi et al. (28)	PDAC	Depletion of gut microbiome leads to reduced IL-10 and IL-17 levels, causing increased IFN-*γ*^+^ CD8 T cell infiltration
	Amy et al. (123)	Colitis-associated tumorigenesis	Mice with *Prevotellaceae* enrichment in the gut microbiome showed higher tumor susceptibility, with tumors showing exhausted CD8 T cell phenotype in the form of PD-1^+^ Lag-3^+^ and PD-1^+^ Tim-3^+^ CD8 T cells
	Fluckiger et al. (118)	Sarcoma	MHC class I restricted IFN-*γ*^+^ CD8 T cells specific for Phage encoded TMP-1 of *E. hirae* cross reacts with tumor specific PSMB4 to provide antitumor immunity.
	Riquelme et al. (15)	PDAC	Long term survivors with enriched *Pseudoxanthomonas, Saccharopolyspora and Streptomyces* have increased CD3^+^, CD8^+^ and Granzyme^+^ T cells intratumorally
**Th17 cells**	Daillère et al. (124)	Sarcoma	*E. hirae* systemically translocates upon CTX treatment and induces pTh17 response characterized by IFN-*γ*^+^ IL17^+^ cells and CCR6^+^CXCR3^+^CD4^+^ T cells, leading to a Th1 antitumor response
**T_regs_**	Le Noci et al. (30)	Metastatic melanoma	Local commensals in the lung induce IL-10 secreting Foxp3^+^ T_regs_ which suppress CD8 T cell and NKT cell infiltration
**γδ T cells**	Jin et al. (26)	lung	Commensal bacteria induce an immunosuppressive TME by upregulating IL-17 producing V*γ*6^+^V*δ*1^+^ *γδ* T cells in a Myd88 dependent manner
	Cheng et al. (29)	Metastatic melanoma	Abx treatment leads to impaired IL-17 secretion by local *γδ* T cells in the lung, causing accelerated metastatic melanoma growth
	Daillère et al. (124)	Sarcoma	Intestinal accumulation of *B. intestinihominis* upon cyclophosphamide treatment led to increased Tc1 and Th1 immune response systemically, along with IFN-*γ*^+^ *γδ* T cells intratumorally
**Follicular T helper cells (T_FH_)**	Roberti et al. (125)	Proximal colon cancer	Apoptotic IECs can induce T_FH_ infiltration and reduce tumor growth by stimulating IL-1β and IL-12 secretion from DCs under the control of ileal microbiota
**Th9, Tc9 cells**	Almeida et al. (73)	melanoma	Increased tumor growth in abx treatment due to gut microbial dysbiosis along with decreased IL-9 producing CD4 and CD8 T cells intra-tumorally
**Mucosal associated Invariant T cells (MAITs)**	Li et al. (126)	CRC	Tumor infiltrating MAIT cells can be specifically activated by *F. nucleatum* through TCR ligation, upon which they express CD39

### Dendritic Cells

Dendritic cells residing in the lamina propria are one of the first immune cells to encounter microbes, recognizing them through PRRs and acting as antigen presenters for the adaptive immune system. Paulos et al. found that the TLR4 mediated signaling *via* the gut commensals led to activated dendritic cells which potentiated the anti-tumor effects of adoptively transferred CD8 T cells post lympho-ablation with radiotherapy in melanoma mice models (119). On the other hand, butyrate production by the gut microbes can inhibit antigen presentation by dendritic cells leading to decreased anti-tumor CD8 T cell response post radiotherapy in models of melanoma and lung cancer (120). The same authors were also able to demonstrate the ability of gut microbial modulation with vancomycin to stimulate IL-12 secretion by CD8*α*^+^DCs and improve cytotoxic T cell response against lung and cervical cancer models expressing HPV E7 proteins (127). Increased IL-1 and IL-12 secretion, on exposure to gut commensals, by tissue resident CD103^+^ DCs is essential for inducing activated cytotoxic T cell response and mediating the immunomodulatory effects of chemotherapy as well as immunotherapy in multiple tumor models (12, 125, 128).

### Natural Killer T Cells

NKT cells are members of the innate immune system which share homology with T cells, have cytotoxic activity and are intimately involved in anti-cancer immune responses (129). Ma et al. have observed that gut bacteria, which metabolize primary bile acids into secondary bile acids, impede immunosurveillance of liver tumors by downregulating accumulation of CXCR6^+^ NKT cells in a CXCL16 (C-X-C Motif Chemokine Ligand 16)-dependent manner. Modulation of the gut microbiome using vancomycin unshackled the NKT cell mediated anti-tumor immune response (35).

### Myeloid Cells

Tumor associated macrophages (TAMs) are M2 polarized macrophages which produce chemokines and cytokines in the TME to suppress cytotoxic T cell response and promote tumor growth and metastasis (130). Microbial dysbiosis has been shown to induce M2 phenotype through cathepsin K (CTSK) mediated TLR4 signaling, thus creating an immunosuppressive milieu and promoting colonic tumor growth (121). Similarly, intra-tumoral bacteria have been shown to stimulate TLR2 and TLR5 in PDAC TME, thus polarizing macrophages towards an M2 phenotype and consequently inducing a tumor-permissive Th2 phenotype (53). *Fusobacterium* species have been shown to accelerate CRC progression by manipulating the innate immune system to induce MDSCs and TAMs in the TME, and thereby suppressing T cell response (49, 50). Rutkowski et al. have noticed that TLR5-dependent commensal bacteria drive malignant progression at extra-mucosal locations by increasing systemic IL-6, which drives mobilization of MDSCs. MDSCs cause *γδ* T cells to secrete galectin-1, thus attenuating anti-tumor immunity in sarcoma mice models (32).

### CD4^+^ and CD8^+^ T Cells

As mentioned above, IFN-*γ* secreting CD4+ and CD8^+^ T cells specific for microbial epitopes have been found to be associated with good clinical outcomes in PDAC. It appears that gut microbiome modulates anti-cancer adaptive immune response as depletion of gut microbiome in mice bearing pancreatic tumors led to increased IFN-*γ* secreting CD8 T cells as well as decreased IL-10 and IL-17 secreting T cells (28). Intriguingly, interaction of microbes with CD4+ and CD8^+^ T cells has been shown to impact the cancer response to immunotherapy and chemotherapy as well. While studying the response of sarcoma tumors to cyclophosphamide, Daillère et al. found that *E. hirae* translocated from the small intestine to secondary lymphoid organs and increased the intra-tumoral CD8/Treg ratio; while *B. intestinihominis* accumulated in the colon and promoted the infiltration of IFN-*γ*-producing *γδ*T cells in cancer lesions (124). Similar observations were reported in the context of *B. fragilis* specific T cells modulating response to anti-CTLA4 immunotherapy to sarcoma (124, 128) and CD4^+^ T induced by *A. muciniphila* modulating response to the anti-PD-1 therapy to NSCLC (12).

Gut microbial dysbiosis associated with colitis can also shape tumor susceptibility through induction of T cell exhaustion. In a model of colitis-induced tumorigenesis, it was observed that FMT from mice developing higher tumor burden was able to induce increased tumor growth in GF mice as compared to FMT from mice with lower tumor burden. This effect was lost when FMT was done in Rag−/− and CD8−/− mice. The authors noted that intra-tumoral T cells in mice with higher tumor burden showed an exhausted phenotype with increased PD-1^+^ Lag-3^+^ and PD-1^+^ Tim-3^+^ CD8 T cells. Differential bacterial signatures in the form of enrichment of *Prevotellaceae* in mice with higher tumor burden and enrichment of *Anaeroplasmataceae* in those with lower tumor burden were also observed (123).

### Follicular T Helper Cells

Follicular T helper cells (T_FH_), which are abundant in mucosal lymphoid tissue and tumor draining lymph nodes, are another important component of the adaptive immune system which are involved in the cancer–microbiome crosstalk. Investigating the effects of oxaliplatin chemotherapy on proximal colon cancer, Roberti et al. (125) showed that apoptosis of intestinal epithelial cells (IECs) in the ileal crypts could induce T_FH_ infiltration into proximal colonic tumors in an IL-1R and IL-12 dependent manner and reduce tumor growth. This effect was dependent on the local ileal microbiota, with some bacteria acting as immunogenic agents (*B*.  *fragilis*, a non-enterotoxigenic species, *E*.  *ramosum* and *A*. *onderdonkii*) while others acting as tolerogenic agents (*F*. *nucleatum*, *P*. *clara*, *B*. *uniformis* and *S*. *gallolyticus*). The authors were also able to harness the immunogenicity of the apoptotic ileal cells to create an immuno-stimulatory vaccine against murine colon cancer. However, ileal microbiota were needed in an adjuvant capacity as the vaccine lost its efficacy under GF conditions (125).

### *γδ* T Cells

*γδ* T cells, which express TCRs but are not dependent upon MHC molecules for antigen recognition, and hence can mount an immune response against diverse antigens, are thought to serve as a bridge between the innate and adaptive immune response (131). Jin et al. showed that commensal bacteria, through Myd88 dependent signaling, induced IL-17 producing V*γ*6^+^V*δ*1^+^ *γδ* T cells in genetically engineered KP (Kras*^LSL-G12D^*; p53*^flox/flox^*) models of lung adenocarcinoma, thus leading to an immunosuppressive TME. Ablation of microbiome was able to rescue anti-tumor response through upregulation of IFN-*γ* in the resident *γδ* T cells. The authors identified taxa like *Herbaspirillum* and *Sphingomonadaceae* which were enriched in tumor bearing lungs while taxa such as *Aggregatibacter* and *Lactobacillus* were enriched in healthy lungs (26). Interestingly, a completely opposite dynamic was observed in models of pulmonary melanoma metastases, where broad spectrum antibiotics impaired a functional *γδ* T cell induction and IL-17 production, leading to accelerated pulmonary metastases (29), again stressing the highly context specific microbiome-immune interaction.

### IL-9 Producing T Cells

As noted earlier, the gut microbiome has also been shown to be essential for IL-9 producing Th_9_ and Tc_9_ cells in the colonic lamina propria. Almeida et al. noticed that GF mice have decreased expression of IL-4 and TGF-*ß*, thereby reducing Th9 cells and leading to increased subcutaneous melanoma tumor growth. FMT from conventional mice into GF mice restores IL-9 production and decreases tumor growth (73). Another evidence for microbial modulation of Th_9_ cells is seen in squamous cell carcinoma (SCC), where Miao et al. found that Th_9_ expanded on exposure to Staphylococcal enterotoxin B (ETB) along with SCC antigens and affected increased cancer cell apoptosis. Mechanistically, SEB significantly increased the levels of signal transducer and activator of transcription 5 phosphorylation (STAT5) and the expression of histone deacetylase-1 (HDAC1) and PU.1 in CD4+ T cells, leading to increased IL-9 secretion (132).

### Mucosal Associated Invariant T Cells

MAITs are characterized by a semi-invariant TCR which recognizes non-peptide epitopes in a major histocompatibility complex class I-related protein (MR-1) dependent manner (133). In CRC, increased infiltration of MAITs is observed, however, IFN-*γ* production is reduced (134), and this increased infiltration correlates with poor survival (135). Analyzing the MAITs from human CRC samples, Li et al. were able to show that *F. nucleatum* in CRC tissue can specifically activate MAITs in a TCR-dependent fashion, leading to expression of CD39 (126). As MAITs can secrete cytotoxic cytokines and modulate anti-tumor immune (133), this study indicates that MAITs could emerge as another potential avenue for targeting the microbial-cancer crosstalk.

## Mechanisms of Microbiome-Immune Crosstalk During Cancer Initiation and Progression

**Figure 1:**
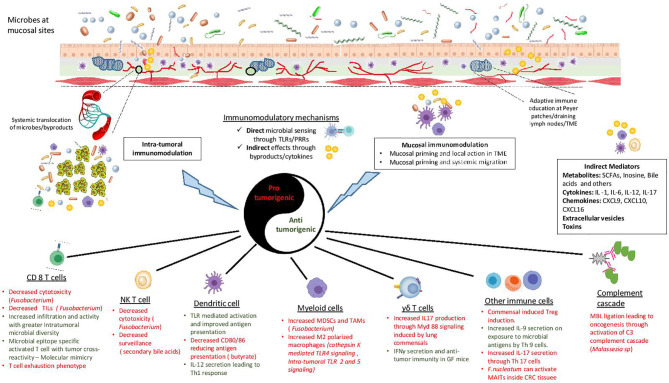
Mechanisms of microbial immunomodulation during tumor progression. Mucosal microbes can modulate the immune system locally or after translocating to the sites of growing tumors. Moreover, they are able to transmit their influences to distant sites using mediators like metabolites, cytokines, chemokines, toxins and vesicles. Microbes can either interact directly with immune cells or provide indirect adjuvant cues for immunomodulation. The consequent inflammation can be either pro-tumorigenic or anti-tumorigenic, with a diverse range of effects on the innate and the adaptive immune system. TLR, Toll like receptor; PRR, pattern recognition receptor; TME, tumor microenvironment; SCFA, short chain fatty acid; TIL, tumor infiltrating lymphocyte; NKT cell, natural killer T cell; MDSC, myeloid derived suppressor cell; TAM, tumor associated macrophage; MBL, mannose binding lectin; MAIT, Mucosal associated invariant T cells.

With improved tools, recent years have seen an increase in our understanding of the mechanism(s) by which microbes (bacteria, virus and even fungus) can alter the immune TME and affect cancer progression (136). Two main mechanisms are often considered to describe this interaction: 1. Microbes influencing anti-tumor effectors directly by serving as antigens 2. Indirect influence by providing adjuvant cues through secreted by-products or inducing cytokine secretion. Following is the description of a few such mechanisms (**Figure 1**):

### Direct Antigenicity

The concept of molecular mimicry, where microbial antigens sharing homology with host antigens induce self-reacting adaptive immune cells, is central to the pathogenesis of autoimmune diseases. In the setting of cancer, numerous microbial derived epitopes resembling tumor antigens were already known, therefore it was hypothesized that molecular mimicry could play a role in anti-tumor immunity as well (137). Actualizing this hypothesis, Fluckiger et al. (118) demonstrated that T cells specific for an epitope of *E. hirae* present in the bacteriophage-encoded tail length tape measure protein 1 (TMP1) could cross react with a sarcoma specific peptide belonging to the proteasome subunit beta type-4 (PSMB4) which improved the anti-tumor immune response observed on treatment with cyclophosphamide. The authors also found that advanced renal and lung cancer patients with detectable fecal TMP at diagnosis exhibited prolonged overall survival after therapy with immune checkpoint inhibitors targeting PD-1. Extending the scope of this hypothesis, they were also able to identify bacterial epitopes of the gut microbiota with significant homology to naturally processed melanoma specific antigens, hinting that the gut microbiome could be a reservoir for numerous antigens capable of mimicking unique tumor epitopes and hence an untapped avenue for designing tumor specific immunotherapeutic strategies (118). This cross-reactivity of microbes with tumor antigens influences anti-cancer immunotherapy as well, as CD4^+^ and CD8^+^ T cells specific for microbial epitopes are necessary for the efficacy of anti-PD1 and anti-CTLA4 therapies (128, 138). The antigenicity of commensal viruses can also play a role in modulating the anti-tumor adaptive immune response. For *e.g.* naturally occurring CD 8^+^ T cells primed by the viral E7 peptide against low-risk *β*-HPV viruses occur in the skin. These T cells can provide immunity against squamous cell carcinoma by inducing a strong immunoselection against high-risk HPV positive dysplastic keratinocytes (139).

Intriguingly, Lu et al. (140) observed an increased degranulation and cytotoxic CD8^+^ T cell response on incubation of PBMC cells from healthy human volunteers with fecal bacteria from CRC patients when compared to fecal bacteria from healthy volunteers. This effect required the presence of antigen presenting cells like monocytes and B cells and was absent when CD8 T cells alone were cultured with the gut microbes. Although in an *ex vivo* setting, this observation again lends to the notion that there are certain bacterial epitopes, even in dysbiotic stool of cancer patients, which harbor the potential to energize the adaptive immune cells to mount an anti-tumor immune response (140).

Another interesting aspect of microbial antigenicity is their ability to give rise to tumor neo-antigens. Viral genome open reading frames (ORFs) are a known source of neo-antigens for virus-associated tumors like human papillomavirus (HPV)-related cervical or oropharyngeal cancer, Merkel cell polyomavirus (MCPyV)-related Merkel cell carcinoma (MCC) and Epstein-Barr virus (EBV)-related head and neck cancers (141). Indeed, long term survivors of pancreatic cancer were found to harbor significantly greater high quality neo-antigens which shared homology with human infections derived class-I restricted epitopes as compared to short-term survivors (142). As tumor neo-antigens have assumed a central role in the search for avenues to improve immunotherapy as well as identify markers predicting immunotherapy response (141), it will be interesting to observe how microbial epitopes will be interrogated to modulate the tumor immune-surveillance.

### Indirect Adjuvanticity

Direct interaction between the microbes and immune effectors is not a necessity, as adjuvant signals can lead to immunomodulation as well. These signals can be sent in the form of various microbial secreted products like metabolites, toxins and vesicles or cytokine secretion induced through manipulation of host cells. These mechanisms are discussed in more detail in the following section.

## Role of Microbial Metabolites and Other Mediators in Modulating Cancer–Immune Interaction

Microbes residing in our body are dynamic metabolic factories. They process nutrients derived from the host and generate a wide range of secreted metabolites. These metabolites or byproducts can have direct effects on tumors or exert their effects indirectly through immunomodulation. The classic example of a direct effect is the carcinogenic effect of colibactin produced by certain colibactin-producing *Escherichia coli*. Colibactin can alkylate DNA on adenine residues (143) and induce double strand breaks in cultured cells (144) which results in a distinct mutational signature, same as the one found in human colorectal cancer patients (145). New evidence also suggests that the microbiome can dictate oncogenic effects of genetic mutations like Tp53 through secretion of the bacterial metabolite gallic acid, hinting at an epigenetic role of microbiome in modulating plasticity of cancer mutations (5). In breast cancer, microbial metabolites like lithocholic acid (146), SCFAs (147), cadaverine (34) and de-conjugated estrogens (148) have been shown to contribute to proliferation, stemness and aggressiveness by having a profound impact on mitochondrial metabolism. While numerous studies have shown the direct effects of microbial byproducts on tumors, there is a keen interest in unravelling the indirect impact on cancer immunosurveillance as well (**Table 3**).

**Table 3:** Immunomodulatory effects of microbial metabolites in tumor progression.

Metabolite	Author	Study type	Cancer	Microbe identified	Comments
**Deoxycholic acid (DCA)**	Yoshimoto et al. (149)	Mouse	HCC	*Clostridium* cluster XI, including *Clostridium sordellii*	Promotion of SASP in HSCs, causing local inflammation
**Fecal and plasma short chain fatty acids**	Nomura et al. (150)	Human (52 patients with solid organ tumors initiating ICI therapy)	Multiple	NA	Fecal acetic, propionic, butyric and valeric acid and plasma isovaleric acid associated with longer PFS on ICI
**Butyrate**	Uribe-Herranz et al. (120)	Mouse	Melanoma, lung cancer	Vancomycin-sensitive gut microbes	Inhibition of antigen cross priming of adoptively transferred CD8 T cells by CD11c^+^ dendritic cells post radiation therapy
**Secondary bile acids**	Ma et al. (35)	Mouse	Primary and metastatic HCC	Vancomycin-sensitive bile acid metabolizing bacteria	Primary bile acids necessary for CXCL16 dependent NKT immune surveillance of HCC
**Inosine and its metabolites, xanthine and hypoxanthine**	Mager et al. (151)	Mouse	Colon cancer	*Bifidobacterium pseudolongum, Lactobacillus johnsonii, and Olsenella species*	Bacterial translocation on ICI initiation led to inosine production and activation of Th1 immune response intratumorally in a inosine-A_2A_R-cAMP-PKA dependent manner
**Anacardic acid**	Frankel et al. (152)	Human (39 patients with metastatic melanoma on ICI therapy)	Metastatic melanoma	Stool of responders enriched with *Bacteroides caccae*	ICI responders had elevated fecal anacardic acid as compared to non-responders.
**Mevalonate and dimethylglycine**	Tanoue et al. (52)	Mouse	Colon	Mix of 11 human gut commensals	Specific IFN-*γ*^+^ CD8 T cells induced systemically associated with increased circulating levels of microbial metabolites
**Glycero-phospholipid and sphingolipid metabolism**	Lv et al. (153)	Mouse	Colon	*Bacteroides acidifaciens*	GQD, a probioitic, modulates gut microbiome and metabolome and synergizes with anti-PD1 immunotherapy

### Short Chain Fatty Acids

It is well documented that SCFAs have histone deacetylase (HDAC) inhibiting activity (154) which can regulate innate immunity pathways, controlling myeloid cell differentiation and inflammatory response mediated by TLR- and IFN-inducible gene expression (155). Interestingly, Nomura et al. found that in patients with advanced/metastatic solid cancer tumors undergoing anti-PD1 therapy, there was an enrichment of short chain fatty acids (SCFAs) in the stool of responders compared to non-responders (150). This may hint at a possible epigenetic role for SCFAs in immunotherapy efficacy, especially when one considers that HDAC inhibition can upregulate the expression of PD-L1 and PD-L2 in melanoma cells and synergize with anti-PD L1 therapy (156). On the other hand, serum butyrate levels limit the anti-tumor efficacy of anti-CTLA4 therapy in metastatic melanoma by restraining the up-regulation of CD80/CD86 on dendritic cells and ICOS on T cells, accumulation of tumor-specific T cells and memory T cells (157). As already described, butyrate secreted by the gut microbes has also been shown to inhibit DC mediated antigen presentation in models of radiation induced tumor necrosis leading to inhibition of cross-priming of tumor specific CD8 T cells (120).

### Bile Acids

Primary bile acids are essential for maintaining a CXCL16-dependent NKT cell immunosurveillance of metastatic liver disease; however, their metabolism into secondary bile acids leads to impairment of anti-tumor immunity (35). Deoxycholic acid (DCA), a secondary bile acid, can induce gut dysbiosis, alter the intestinal barrier function, recruit M2 macrophages and promote intestinal carcinogenesis in models of spontaneous intestinal tumorigenesis (158). Microbial DCA has also been implicated in the pathogenesis of HCC. Dietary or genetically induced obesity results in gut microbial dysbiosis which in turns increases the levels of DCA, resulting in a senescence associated secretory phenotype (SASP) in hepatic stellate cells (HSCs), leading to local inflammation and tumorigenesis (149). Loo et al. further characterized this interaction by demonstrating that lipoteichoic acid from the gram-positive gut microbes activated TLR2-dependent prostaglandin E2 production to suppress adaptive immunity in the TME while also potentiating the DCA mediated SASP in obese mice (159).

### Inosine and Its Metabolites

Inosine, a purine metabolite, was recently shown to be an important modulator of response to immune checkpoint blockade (ICB) therapy in multiple tumor models (151). The authors identified three bacterial species—*Bifidobacterium pseudolongum, Lactobacillus johnsonii, and Olsenella species*, which translocated into the systemic circulation on ICB initiation, produced inosine or its metabolite hypoxanthine, and induced Th1 differentiation and effector functions through inosine-A_2A_R-cAMP-PKA pathway. This immune-stimulatory effect though was context dependent, as it required co-stimulation in the form of exogenous IFN-*γ* as well as IL-12 binding on T cells (151). Another recent study elucidated the role of inosine as an alternative carbon source for T cell metabolism in a glucose restricted setting, thereby assisting proliferation and differentiation of T cells and improving response to ICB, supporting the idea that inosine metabolism could be playing an integral part in anti-tumor immunity (160). These are intriguing findings when juxtaposed with the fledgling field of adenosine receptor (A_2A_R) blockade for improving cancer immunotherapy (161, 162) and indicate that the gut microbiome might influence how this therapy evolves.

### Correlative Evidence Between Microbial Metabolites and Tumor Immunity

The systemically induced, tumor suppressing IFN*γ*^+^ CD8 T cells observed by Tanoue et al. were found to be associated with increased fecal and circulating bacterial metabolites like mevalonate and dimethylglycine (52). A naturally occurring xenobiotic, anacardic acid, which can activate macrophages (163), induce neutrophils extracellular traps (NETs) (164) and has previously been shown to potentiate anti-cancer immunity (165), was found to be enriched in the stool of metastatic melanoma patients who responded to ICI therapy as compared to non-responders (152).

### Other Modes of Gut Microbiome-Immune Communication

While metabolite-mediated interaction is the predominant mode purportedly involved in microbe–host crosstalk, cytokine secretion instigated by the gut microbiome can also exert immunomodulatory effects during tumor progression. Microbiota-dependent IL-17 secretion has been shown to modulate the immune TME in PDAC (28), ovarian and breast cancer (32) as well as lung cancer (26, 29). Moreover, systemically induced TLR5 dependent IL-6 secretion can also drive MDSC recruitment and suppress anti-tumor immunity (32). IL12 is another important cytokine which is released by DCs activated upon microbial interactions and leads to induction of a Th_1_ response (12, 125).

Gut microbes and their host can also interact through extracellular vesicles (EVs). Microbial EVs transport cellular signaling molecules, metabolites or antigenic proteins which can trigger inflammatory responses and immunomodulation under homeostasis as well as pathological processes (166). Moreover, EVs serve important functions in tumor development, providing proliferative signals, enabling metastasis and even inducing immune escape through checkpoint molecules (167, 168). A recent study showed that EVs derived from a commonly used probiotic, *Lactobacillus rhamnosus GG*, exert direct anti-tumor effects on hepatic cancer cell growth (169). Owing to the lack of exploratory studies, it remains to be seen if EVs are one of the instruments through which the microbiome manipulates the immune system to affect oncogenesis.

Another intriguing interaction of the gut microbiome with the immune system is through their binding with NLRP proteins to activate inflammasomes. In homeostatic states, commensal bacteria activate NLRP3 inflammasomes to produce IL-18, which is critical for maintaining intestinal barrier integrity and preventing microbial dysbiosis (170). Inflammasome activation through commensal bacteria can also modulate adaptive immune response and provide immunity against viral pathogens (171). Inflammasome activation can modulate tumor growth across multiple cancers like colon, breast and pancreas (172–174). Interestingly, in mice models of melanoma with defective Ubiquitin Protein Response (UPR), there was upregulation of inflammasome components, alteration of gut microbial composition with enrichment of species like *B. rodentium*, improved anti-tumor immunity through increased DCs recruitment and improved immunotherapy efficacy (27). Further evaluation of NLRP ligation might reveal novel mechanisms for microbiome-mediated immunomodulation in the TME.

Microbial genotoxicity might play a role in anti-tumor immunity as well. Analysis of sputum samples from patients with lung cancer and healthy individuals showed an increased prevalence of leucocytes with chromosomal aberrations in patients with cancer (175). There was a significant difference in the beta diversity of the sputum microbial composition, with genuses like *Atopobium* and *Treponema* decreasing significantly and *Bergeyella* increasing significantly in cancer patients. Reduction in species from genus *Atopobium* and increase in *Alloprevotella* species correlated with higher levels of chromosomal aberrations in sputum donors (175). On the other hand, oral administration of *Lactobacillus johnsonii* was able to alleviate lymphocytic as well as systemic genotoxicity in Atm^−/−^ mice models of Ataxia-telangiectasia, thereby decreasing the development of lymphoma (176). However, more mechanistic studies evaluating the role of genotoxic effects of microbes on the immune cells in the TME are needed for establishing their significance in the tumor–microbiome–immune trialogue.

## Do Microbes Act in Strictly Restricted Compartments?

When tumors develop in sites naturally colonized with commensal microbes, such as colon, lung or the skin, we expect a local immunomodulatory effect of the microbiome in the TME through their interaction with mucosal dendritic cells and lymphocytes. However, emerging evidence now firmly supports the fact that the microbiome can determine cancer immunosurveillance in traditionally sterile sites like pancreas, breast and the urothelium. Detection of intra-tumoral microbial DNA and metabolites is fraught with challenges owing to the miniscule microbial load, contamination from processing procedures and the huge amount of host DNA which can confound any results. Overcoming these barriers, Nejman et al. (177) sequenced 1,526 human samples from seven different tumor types (melanoma, breast, bone, brain, pancreas, ovary, lung) and comprehensively identified unique metabolically active live bacterial species inside the tumor cells in different tumor types (177). This finding again compels us to reconsider the traditional paradigm of certain tumor-bearing sites being inaccessible to microbes. Direct bacterial translocation due to iatrogenic causes like endoscopy or secondary to gut mucosal inflammation may be responsible for some of these findings. There is also evidence of increasing expression of adhesion molecules binding to bacterial cell wall components upon tumor growth which might facilitate bacterial homing (33, 46). In all these situations, the presence of microbes in the TME should encourage local immunomodulation inside the tumors.

Based on studies where ablating the gut microbiome using poorly absorbable antibiotics still affected tumor growth through immunomodulation in sites such as pancreas (15, 28), an intriguing scenario emerges, with the possibility of distant effects being exerted by mucosal commensals. One possible explanation for this effect could be decreased bacterial translocation from the gut. However, bacteria translocation may not be a necessity for this immunomodulation. Bacterial antigens can migrate across a disrupted mucosal barrier into the lymphatic system and eventually activate PRRs at distant sites (178). The plethora of metabolites, toxins, and cytokines being secreted by these commensals can also serve as distant second messengers.

Moreover, it might be possible that locally primed mucosal immune cells migrate into the systemic circulation and execute tumor modulatory effects at remote sites. Mucosal DCs in the colon, loaded with microbial antigens, can be found at tumor draining lymph nodes and influence anti-tumor immunity (12, 125). Functionally plastic adaptive immune cells like Th17 cells, which can get primed at mucosal sites and exert effects at distant sites, are already known in the setting of autoimmune diseases (179, 180); however, their significance in cancer immunosurveillance is yet to be elucidated.

Arguing against the migration of mucosal immune cells, Tanoue et al. found that oral inoculation of specific microbes induced distinct populations of IFN*γ*^+^ CD8 T cells locally and systemically. The local population was characterized by higher expression of TCR V*β*6^+^ and V*β*8^+^ subsets. However, in the setting of tumor implantation, there was a distinct enrichment of tumor infiltrating CD8 T cell population characterized by the V*β*13^+^ TCR subset (52). The presence of distinct TCR subsets in mucosal and systemic T cells suggests that effector T cells educated by the mucosal microbes operate in restricted compartments and do not translocate systemically to distant sites. In another study supporting a more compartmentalized immunomodulation during lung adenocarcinoma progression, Jin et al. showed that commensal bacteria induced a locally proliferating population of IL-17 producing RORγt^+^ γδ T cells. These cells were of local origin, as demonstrated on reconstitution of irradiated KP-CD45.1 mice with donor KP-CD45.2 mice marrow, wherein the ROR*γ*t^+^ *γδ* T cells proliferating inside the tumor bearing lungs were still of the recipient origin while donor-derived V*γ*6^+^V*δ*1^+^ T cells were virtually absent, indicating that the microbe-modulate immune population maintained their mucosal sovereignty, with no recruitment from the circulating immune cells (26).

Interestingly, in the study by Riquelme et al., the authors found that although the gut microbial species could not fully account for the intra-tumoral pancreatic microbial species on the basis of direct microbial translocation, the gut microbial composition affected the tumor microbiome composition, hinting at the possibility that the gut microbiome can remotely modulate not only the immune milieu but also the intra-tumoral microbiome itself (15). Further studies are required to delineate the site of this microbiome-immune crosstalk and identifying the putative compartments for targeting this interaction.

## Beyond Bacterial Pathobionts—Fungome and Virome in the Spotlight

Microbiome is sometimes taken to be synonymous with bacteriome. However, within the diverse ecosystem that is the human body, commensal fungi, viruses, and protozoans have carved out their own niches. As the field of microbiome studies has expanded, it has recently become evident that these overlooked citizens can have an important role in tumorigenesis and immunosurveillance. For instance, *Malassezia* sp, which commonly inhabit the skin and mucosal tissue, were surprisingly demonstrated to be enriched in human and mice PDAC tumor samples (55). In the TME, *Malassezia* ligated the mannose binding lectin (MBL) receptor on the innate immune cells to induce complement protein C3 dependent cascade, which resulted in accelerated oncogenesis (55). Epidemiological studies have also shown evidence of fungal dysbiosis in other neoplastic states. The phylum *Glomeromycota* was found to be depleted in tumor tissues from patients with oral cancer as compared to adjacent normal tissue (181), while in a separate study, differential abundance of *Candida albicans*, *Rothia mucilaginosa*, and *Schizophyllum commune* was observed between oral washes from individuals with and without HNSCC (182). Fungi from the phylum *Chytridiomycota* and *Glomeromycota* were also observed to be enriched in colorectal adenomas as compared to healthy tissue (183).

Viral particles are thought to be the most abundant microbes in the biosphere, outnumbering even the bacteria (184, 185). A number of human cancers have been shown to be dependent upon viral replication and integration into the host genome, including cervical squamous cell carcinoma on HPV infection, numerous EBV induced HNSCC, Merkel cell cancer, *etc*. In the context of the gut microbiome, the viral component is dominated by bacteriophages (186). Through their ability to transform bacterial cells, they can confer novel properties like antibiotic resistance, toxin production, control quorum sensing and virulence and neoantigen expression, all of which are central to the oncogenic activity of bacteria (187). Their role in the microbiome–cancer dynamic is poorly understood due to challenges in virome sequencing caused by the absence of phylogenetically conserved regions within different viruses and our inability to assign the majority of the sequenced viruses to specific clades. However, recent studies, such as the one by Fluckiger et al. (118), underscore the important role commensal bacteriophages can play in the anti-tumor immune response. Interestingly, phages belonging to *Siphoviridae* and *Myoviridae* have been shown to correlate with abundance of Fusobacterium and reliably differentiate CRC and adenoma patients from healthy volunteers (188). As we gain more insights into the molecular mechanisms of phage–bacteria–tumor interactions, we will be able to decode the exact nature of this relationship and its importance for tumor dynamics. **`**

## Implications for Cancer Immunotherapy

Earliest evidence suggesting that microbes could be utilized for cancer immunotherapy stems from studies wherein sarcoma regression was induced by direct injection of *Streptoccocus* and *Serratia* species into tumor (189). Along similar lines, anti-tumor efficacy of Bacillus Calmette–Guerin in bladder cancer supported this possibility (190). In recent years, the concept that microbes can modulate immune response to cancer has again come to forefront. Pioneering studies by Iida et al. have demonstrated that commensal bacteria in gut potentiate the anti-tumor effects of CpG-oligonucleotide immunotherapy and oxaliplatin chemotherapy by modulating function of myeloid cells (122). This report was followed by evidence that even the immunomodulatory effects of cyclophosphamide are shaped by the gut microbes and that the gut microbes promote induction of Th-1 type immunity and infiltration of Th-17 cells in the TME (191). Since these initial reports, subsequent studies have described the role of gut commensals like *Bifidobacterium* (138) and *B. fragilis* (128) in modulating the efficacy of anti-PDI and anti-CTLA4 respectively. In the current section (also **Table 4**), we have discussed the key studies which have added to our current understanding of the ability of gut microbiome and microbiome at other sites to influence the efficacy of immunotherapy:

**Table 4:** Role of gut microbiota in immunotherapy efficacy and toxicity.

Immunotherapy	Author	Study type	Cancer	Associated microbes/phyla	Findings
**Anti-PD-1/PD-L1**	Sivan et al. (138)	Mouse	Melanoma	*Bifidobacterium*	Augmented DC mediated cross priming of CD8 T cells to induce tumor-specific cytotoxic response
	Gopalakrishnan et al. (13)	Human gut microbiome avatar mice	Melanoma (43 patients, 30 responders (R), 13 non-responders (NR)	*Faecalibacterium* (R), Bacteroidales (NR)	Tumor samples from R had increased CD8 T cell infiltration while *Faecalibacterium* was enriched in stool. FMT from R into GF mice showed decreased tumor growth with increased adaptive immune response in TME.
	Routy et al. (12)	Human gut microbiome avatar mice	NSCLC (100 patients)	*A. muciniphila*	Stool of ICI responders were enriched with *A. muciniphila*. Oral supplementation of *A. muciniphila* to non-responder avatar mice improves PD-1 response by recruiting CCR9^+^CXCR3^+^CD4^+^ T lymphocytes
	Matson et al. (192)	Human gut microbiome avatar mice	Metastatic melanoma (42 patients, 16 responders, 26 non-responders)	*Bifidobacterium longum, Collinsella aerofaciens*, and *Enterococcus faecium*	Several bacterial species enriched within stool of R. FMT from R into GF mice or Taconic mice led to greater tumor control than NR FMT, with increased CD8 T cell in TME.
	Sethi et al. (28)	Mouse	PDAC	NA	Broad spectrum antibiotics depletion of gut microbiome improves efficacy of PD-1
	Pushalkar et al. (53)	Mouse (KC mice, spontaneous PDAC model)	PDAC	*Bifidobacterium pseudolongum*	Gut microbial ablation upregulates PD-1 expression on CD4^+^ and CD8^+^ T cells, improving efficacy of anti PD-1 therapy
	Anker et al. (193)	Mouse	Prostate	Uropathogenic *E. coli*, CP1	Exogenously administered CP1 causes immunogenic cancer cell death, improving Th1 response and anti-PD1 efficacy
	Tanoue et al. (52)	Mouse	Colon, Melanoma	11-mix of human gut commensals	Increased anti- PD-1 efficacy along with higher IFN-*γ*^+^ PD-1^high^CD103^−^ CD8^+^ T cells, GrB^+^IFN*γ*^+^ CD8 T cells and MHCII^+^CD11c^+^ DCs Toxicity: Colonization with 11-mix was also able to decrease colitis upon treatment with PD-1 or CTLA4 therapy
	Zheng et al. (194)	Human	HCC (8 patients with Sorafebin resistant HCC)	*Proteobacteria* – NR *Lactobacillus, Ruminococcaceae* and *Akkermansia muciniphila* - R	Dynamic changes in microbiome during ICI. Proteobacteria enriched in NR stool from week 3 to week 12. R stool enriched with multiple taxa.
	Liu et al. (195)	Mouse	Colon	Improved fecal *Firmicutes/Bacteriodes ratio*,increased *Lachnospiraceae* and decreased *Muribaculaceae*	Bilberry anthocyanin combo, a probiotic, enhances PD-1 efficacy and lymphocyte infiltration in tumors
	Lv et al. (153)	Mouse	Colon	*Bacteroides acidifaciens*	Combination of GQD, a probioitic, with anti-PD1 therapy led to alteration of the gut microbiome and metabolome along with increased immunotherapy efficacy, systemic and tumor CD8 T cells as well as IFN-*γ* production
**Anti-CTLA4**	Vetizou et al. (128)	Mouse	Sarcoma	*Bacteroides fragilis*	*B. fragilis* gavage to GF mice restores efficacy of anti-CTLA4 immunotherapy *via* maturation of intra-tumoral DCs leading to an enhanced Th1 immune response
	Chaput et al. (196)	Human	Metastatic melanoma (26 patients, prospectively collected stool samples)	*Faecalibacterium*	Baseline gut microbiome enriched with *Faecalibacterium* was associated with increased ICI response, lower peripheral Tregs and higher iCOS induction on CD4^+^ T cells. Toxicity: *Faecalibacterium* enrichment associated with decreased colitis on immunotherapy treatment
	Dubin et al. (197)	Human	Metastatic melanoma (34 patients, prospectively collected stool samples)	*Bacteroidetes*	Increased fecal abundance of the *Bacteroidetes* phylum, as well as microbial genetic pathways involved in polyamine transport and B vitamin biosynthesis, are correlated with resistance to the development of colitis following CTLA-4 blockade
	Coutzac et al. (161)	Human and mouse	Metastatic melanoma	*Faecalibacterium*	*Faecalibacterium* levels correlated with improved immunotherapy response. Serum butyrate levels negatively correlated with anti-CTLA4 response and were associated with decreased sCD25 and ICOS^+^ CD4 T cells.
**Anti-PD1 and CTLA-4 combination**	Frankel et al. (171)	Human	Metastatic melanoma (39 patients)	*Bacteroides caccae, Faecalibacterium prausnitzii, Bacteroides thetaiotamicron, and Holdemania filiformis*	Out of 39 patients, 24 received combination therapy with PD1 and CTLA4. Stool of responders was enriched for specific bacteria (as noted) and had increased fecal metabolites involved in amino acid metabolism
	Salgia et al. (198)	Human	Metastatic RCC (31 patients, 23% receiving combination immunotherapy)	*Akkermansia muciniphila*	*Akkermansia muciniphila* progressively increased throughout the duration of therapy in patients deriving clinical benefit. Greater alpha diversity of stool microbiome correlated with greater clinical benefit
	Mohiuddin et al. (199)	Human	Stage III/IV melanoma (568 patients, retrospective study)	NA	Antibiotics use within 3 months prior to starting ICI associated with worse survival and increased moderate to severe colitis incidence.
**CpG ODN**	Iida et al. (122)	Mouse	Colon, Melanoma	*Lactobacillus* correlates negatively while *Ruminococcus* and *Alistipes shahii* correlate positively with immunotherapy efficacy	Abx-mediated gut microbial depletion decreases TNFα secreting myeloid cells in the TME, thereby reducing the efficacy of anti-IL10R/CpG ODN combination immunotherapy
**Anti-CD47**	Shi et al. (200)	Mouse	Colon	*Bifidobacterium*	Intratumorally accumulated *Bifidobacterium* facilitates anti CD47 immunotherapy by activating STING-dependent type I INF mediated increased DC antigen presentation
**Anti-Tim-3**	Lee et al. (201)	Mouse	Colon, melanoma, ovary	*L. johnsonii, E. hirae*	Abx induced gut dysbiosis retards anti-tumor efficacy of Tim-3 blockade, which can be partially rescued upon oral gavage of specific bacterial

### Anti-PD1/L1 and Anti-CTLA4 Therapy

Antibodies to block PD-1/PDL-1 and CTLA-4 immune checkpoint mechanisms are one the earliest immunotherapeutic strategies which have allowed immunotherapy to enter the mainstream of anti-cancer treatment. The past decade has witnessed seminal research regarding the effect of microbiome on the efficacy and toxicity of ICB strategies.

#### Effect of Gut Microbiome Composition on the Efficacy of Immunotherapy

Elucidating the connection between microbes and efficacy of ICB therapy, Routy et al. (12) who observed that in a cohort of 249 cancer patients [advanced NSCLC (n = 140), RCC (n = 67), or urothelial carcinoma (n = 42)], who received PD-1/PD-L1 mAb, antibiotic therapy was associated with significantly decreased overall survival and disease free survival. Inspired by these findings, they investigated the gut microbiome of 100 NSCLC patients on ICI therapy and found that the stool of responders was significantly enriched with *A. muciniphila* as compared to non-responders. They then used the state-of-the-art Human Gut Microbiome AVATAR mice, where the patient’s gut microbiome was transplanted into mice to yield patient specific AVATAR mice, to investigate the implication of this in a controlled setting. They observed that in the AVATAR mice generated by fecal microbiota transplantation (FMT) from PD-1 non-responders, oral supplementation with *A. muciniphila* restored the efficacy of PD-1 blockade in an interleukin-12-dependent manner and led to the recruitment of CCR9^+^CXCR3^+^CD4^+^ T lymphocytes into mouse tumor beds (12). Two other concurrent studies from Matson et al. (192) and Gopalakrishnan et al. (13) linked the commensals *Bifidobacterium longum*, *Collinsella aerofaciens*, *Enterococcus faecium*, and *Ruminococcaceae* with PD-1 immunotherapy response in melanoma. In the past two years, multiple new studies have observed similar findings where the baseline microbiome or its modulation using antibiotics, probiotics, or FMTs have modulated response to immunotherapy (28, 52, 53, 152, 194, 196, 202). Anker et al. observed an interesting phenomenon while investigating the effects of a uropathogenic *E. coli* (CP1) on immunotherapy response of prostate cancer, where the exogenously administered microbes caused immunogenic cancer cell death, leading to activation of Th1 immune response and potentiating anti-PD1 immunotherapy (193). Microbial metabolites have also been linked to immunotherapy efficacy. For *e.g.* Nomura et al. observed a positive correlation between fecal SCFAs and anti PD-1 therapy (150), while Coutzac et al. found an inverse relation between systemic butyrate levels and anti-CTLA4 therapy (157). Thus, multiple studies have now demonstrated that the gut microbiome composition and function can affect ICB response in cancer.

#### Antibiotic Use and Response to ICB

Studies suggest that antibiotics can have long-standing effects on the gut microbiome composition. Moreover, multiple clinical studies have now demonstrated that use of antibiotics can potentially diminish the efficacy of ICBs in multiple cancer types like melanoma, NSCLC, urothelial carcinoma, and other solid organ tumors (11, 199, 203–208). This effect is seen independent of tumor site, disease burden, and performance status (11), indicating that diminished microbial diversity might be one of the main driving factors behind this decreased effectiveness of ICBs. However, these results have been based on the historical use of antibiotics, and none of the studies concomitantly evaluated the gut microbial diversity before and after antibiotic use. Ambiguity also remains over the effect of prior *vs* concomitant use of antibiotics, with some studies only finding a diminished response to ICBs with prior use (11), while others observing effects with concurrent use as well (12, 207, 208). Further convoluting this notion, preclinical studies from our lab and others have demonstrated that gut microbiome depletion with broad-spectrum antibiotics can in fact energize the anti-cancer immune response and synergize with ICBs in pancreatic cancer (28, 53). These observations are in line with the cancer-specific interactions of the commensal flora with immune system, where the interaction with microbes can push the cancer-immune equilibrium towards either a tumor-promoting or a tumor-inhibiting phenotype. Hence, there is a need for prospectively designed studies which can delineate the exact effects of antibiotics therapy on ICB response in specific tumors, and simultaneously identify the unique microbes or their consortium which affect this response.

#### Probiotics Use and Efficacy of Immunotherapy

Just like antibiotics, pro-biotics can modulate the gut microbial diversity and composition (209). Gut microbiome modulation with probiotics has also shown potential to improve immunotherapy efficacy. Bilberry anthocyanin combo, containing chitosan and low molecular citrus pectin (LCP), was found to enhance anti-PD-1 efficacy against murine colon cancer by improving CD4^+^ and CD8^+^ T cells’ infiltration inside tumors. In conjunction with anti-PD1, the probiotics were able to improve *Firmicutes/Bacteriodes* ratio, enrich Lachnospiraceae, and decrease *Muribaculaceae* in the gut, along with increased levels of fecal butyrate as well as upregulated carbohydrate metabolism pathways (195). Another strategy aimed at improving immunotherapeutic efficacy of anti-PD1 therapy against MSS (microsatellite stable) CRC employed traditional medicinal components in the form of GQD (Gegen Qinlian decoction). Combining GQD with PD-1 therapy decreased the growth of CT26 xenografts through increased CD8^+^ T cell infiltration and IFN-*γ* production. This was accompanied by a phylogenetic as well as functional transformation of the gut microbiome with increased *Bacteroides acidifaciens* levels in the gut as well as upregulation of plasma levels of metabolites represented in the glycerophospholipid and sphingolipid metabolism (153). But again, the relationship of pro/prebiotics with cancer and immune response is very context dependent. For instance, while overall literature points to beneficial effects of many pre-biotics, it has also been demonstrated that dysregulated microbial fermentation of soluble fiber can induce cholestatic liver cancer (210). Moreover, individual prebiotics can interact uniquely with select tumor subsets, leading to differing microbial enrichment and anti-tumor immune response, as observed by Li et al. in pre-clinical models of melanoma and colon cancer (197).

#### Microbial Composition and ICB Toxicity

Studies suggest that the gut microbiome composition not only affects the response to ICBs, it also helps determine untoward effects of ICB treatment, specifically gastrointestinal toxicity. A significant number of patients experience colitis post initiation of ICB therapy (211), which might mandate discontinuation of therapy in some cases. Although the exact mechanism of this inflammation is unclear, multiple candidate mechanisms include disruption of intestinal barrier integrity, change in bacterial diversity and composition, alteration of secreted microbial metabolites *etc* (211). Multiple studies have found evidence of the influence of gut microbiome on ICB induced colitis. Dubin et al. have demonstrated that the presence of bacteria from *Bacteroidetes* phylum is associated with a lower rate of CTLA4-induced colitis in melanoma patients (197). In another study with similar results, baseline gut microbiota enriched in *Bacteroidetes* was protective, whereas the presence of *Faecalibacterium* and other firmicutes was associated with frequent occurrence of ipilimumab associated colitis in patients with metastatic melanoma (196). Interestingly, in a recent study, patients with stage 3 and stage 4 melanoma who had exposure to antibiotics within the last 3 months prior to ICB therapy not only had worse survival outcomes but also had greater incidence of moderate to severe immune mediated colitis (199). Tanoue et al. identified 11 microbial species which when repopulated in GF mice negated the colitogenic side effects of anti PD-1 and anti-CTLA-4 (52). Taken together, these findings suggest that the gut microbiome plays a significant role in determining not only the response to ICBs, but also the toxicity of ICBs.

#### Effect of ICBs on Gut Microbiome Composition

Given the role of immune system in determining gut microbial composition, it is not unexpected that gut microbiome changes in response to the ICB therapy. The same was confirmed in the study by Zheng et al. which evaluated the dynamic transitions in gut microbiome profile of patients with sorafenib resistant HCC undergoing anti-PD1 therapy (194). The authors found that in the stools of non-responders, Proteobacteria increased from Week 3 and became predominant at Week 12; this increase was mainly attributable to enrichment of *E. coli*. The gut microbiome of the responders, meanwhile, showed selective increase in bacteria like *Lactobacillus, Ruminococcaceae* and *Akkermansia muciniphila*. Functional analysis using Kyoto Encyclopedia of Genes and Genomes (KEGG) identified several pathways which were over-represented in the resulting gut microbiome of the responders like cellulose and pectin metabolism as well as methanogenesis pathways, indicating an improved energy metabolism might be contributing to the improved response to ICBs (194). Salgia et al. analyzed sequential stool samples from patients with metastatic RCC on nivolumab or nivolumab+ipilimumab combination and observed that the relative abundance of *A. muciniphila* increased in patients deriving clinical benefit from ICB whereas it decreased in some of the non-responders (198). These findings hint at the existence of host and/or tumor specific microbial regulatory pathways which may attempt to dynamically modulate the resident commensals to facilitate or antagonize the anti-tumor immune response. However, it must be noted that few investigators have also observed a relatively stable gut microbial profile during the course of immunotherapy (13, 152).

### Gut Microbiome and Efficacy of Other Immunotherapeutic Strategies

While most of the data regarding the interaction of gut microbiome with the ICB has been in context of PD-1/PDL-1 and CTLA-4, there have been some studies with respect to other immunotherapeutic strategies as well. CD47 is expressed on tumor cells and presents a ‘don’t eat me’ signal to the macrophages (212), hence enabling the tumor cells to escape phagocytosis. Therefore, anti-CD47 blockade is being investigated in multiple cancers as an immunotherapeutic option; however, studies have reported mixed results (213). Investigating these findings, Shi et al. demonstrated that *Bifidobacterium* accumulated inside colon cancer tumors and stimulated type I interferon signaling in a STING (stimulator of interferon genes) dependent fashion to improve cross priming of antigens inside dendritic cells, which in turn facilitated CD47 blockade dependent immune response (200). T cell immunoglobulin and mucin domain-containing protein-3 (Tim-3) blockade is another approach for anti-cancer immunotherapy which aims at alleviating T cell exhaustion and regulatory T cells in the TME to boost anti-tumor immunity. It was recently shown that gut microbial dysbiosis triggered by antibiotic administration decreased the efficacy of Tim-3 blockade therapy. Oral gavage with wild type mice fecal matter, *Enterococcus hirae* or *Lactobacillus johnsonii* was able to transform the gut microbial composition and restore the efficacy of Tim-3 blockade (201).

## Modulating the Microbiome for Therapeutic Gains

Identification of the intricate interactions between the microbiome, cancer, and the immune system opens up potential new avenues for cancer therapy through microbial modulation. The gut microbiome is the most readily accessible niche in this regard. Antibiotics, probiotics and prebiotics, which are routinely used for this purpose in benign pathologies like infections and diarrheal disorders, are increasingly being tested in pre-clinical models as well as clinical trials (214, 215) to assess their effects on tumor growth and therapies. Other strategies like fecal microbiota transfer, which involve transfer of the entire gut microbiome of healthy individuals to patients and are already FDA approved for conditions like *C. difficile* infections (216, 217), are also promising candidates. Preclinical models also indicate the therapeutic potential of microbial monotherapies like *A. muciniphila* and *B. fragilis* for improving immunotherapy response (12, 128). Apart from the gut microbiome, researchers have also tried to manipulate the lung microbiome using aerosolized antibiotics and probiotics (*Lactobacillus rhamnosus GG*) to improve lung cancer and metastatic melanoma growth in preclinical models (30).

However, microbiome-modulation based clinical strategies still need to be optimized. The human gut microbiome tends to remain stable over long periods of time (218) but is liable to extensive short-term variations based on exogenous or endogenous stimuli. Using strategies like antibiotics or probiotics to modulate the microbiome has the caveat that it will perturb the ‘healthy’ commensals along with their tumorigenic counterparts. Therefore, behavioral strategies which modulate the determinants of microbiome like diet-based interventions could also be investigated for cancer therapy. Another alternative could be targeted therapies against selective microbes. Zheng et al. (219) were able to isolate a commensal bacteriophage from human saliva which selectively suppressed the growth of *F. nucleatum* in the colonic mucosa. Oral or intravenous administration of irinotecan-loaded dextran nanoparticles covalently linked to azide-modified phages in models of CRC inhibited the growth of *F. nucleatum*, allowed proliferation of butyrate-producing *C. butyricum* and significantly augmented the efficiency of first-line chemotherapy treatments of CRC while reducing the side-effects of chemotherapy (219). These therapies give us a roadmap for translational application of microbial modulation strategies as they target selective bacteria while preserving the non-pathogenic commensals. Other focused strategies using narrow spectrum antibiotics, probiotics or prebiotics are finding increasing use in preclinical and clinical settings as we enter a new era of microbiome-based strategies for combating tumor growth (220).

## Challenges in Unravelling the Cancer–Microbiome Axis

While we have made significant progress with respect to the tools and strategies to better understand the reciprocal connection between cancer and microbiome. The foremost challenge in microbiome research in the context of tumors is to define a ‘healthy’ microbiome. The highly context-dependent nature of the gut microbiome limits our ability to define an overarching healthy phenotype and this may explain some contrasting results. For *e.g.* antibiotic use impairs immunotherapeutic response in various cancers but has been shown to synergize with immunotherapy in preclinical models of other cancers. Not only do the microbes experience tumor-specific variations, there are other factors at play including inter-individual variations in determinants of microbiome (221) as well as concurrent therapies involved in care of cancer patients. Furthermore, bacterial pathways share a lot of redundancies and multiple bacteria are capable of performing similar functions (222). Thus, even though we identify a single colony, it becomes important to explore and characterize the whole consortium as well as characterize and identify the metabolic pathways which can modulate the tumor and the immune environment.

The second challenge is the ever-shifting technological landscape in microbiome studies. Identification of microbial colonies is currently being carried out predominantly using 16s rRNA amplicon sequencing or shotgun whole genome sequencing. However, these techniques have certain drawbacks which need to be addressed including lower resolution, substitution errors, variations due to selection of different conserved regions in different techniques, inability to phylogenetically classify viruses by 16s rRNA sequencing and variations in sample preparation and processing, loss of bacterial colonies in shallow depth metagenomics and cost considerations for shotgun WGS (223, 224). Another challenge in studying the effects of microbiome is cultivating mice models where the effects of microbiome on tumorigenesis can be studied without imposing pre-existing constraints. For *e.g.* GF mice have a severely compromised immune response which can be corrected on conventionalization to a certain extent, but leaves the door open for confounding results when studying tumor immune environment. SPF mice have to be treated with unabsorbable broad-spectrum antibiotics to sterilize the gut, which still can have some systemic absorption (*e.g.*, metronidazole) or interfere with important physiological functions of the gut like enterohepatic circulation, introducing an additional variable which may affect our disease of interest independent of the microbial composition. Moreover, recolonization of sterilized gut through FMT does not result in successful engraftment of all the microbes present in donor stool, precluding the analysis of pathologic implications of the entire consortium. An important step in this direction is the development of spontaneous models of gut microbiome dependent carcinogenesis as described by Slowicka et al. In this model, transgenic expression of an epithelial mesenchymal transition protein Zeb2 specifically in the intestinal epithelial cells (IEC) led to microbial dysbiosis, increased colonic permeability, and microbiota dependent invasive CRC development, independent of known tumorigenic mutations (225). This is in contrast to other models of spontaneous CRC with microbiota dependent tumorigenesis. For instance, the GF *Apc^Min/+^*;*Il10*^−/−^ mice, which can form spontaneous colonic tumors upon conventionalization with FMT from SPF mice albeit in a manner dependent on known CRC driver mutations like APC (226).

Finally, we have to be mindful of the fact that association does not equal causation. In the case of the human microbiome, there has been a recent explosion of studies correlating microbial dysbiosis with disease states ranging from autoimmune and inflammatory conditions to neurodegenerative conditions, behavioral disorders and metabolic diseases apart from various cancers. Need of the hour is to evaluate specific commensal–disease interactions and delineate the exact mechanistic pathways which underlie these correlations to sift out the spurious correlations from causal relationships (227).

## Future Directions

While we have gleaned significant new insights into the specific microbiome-immune interactions in the setting of cancer, a lot more work remains to be done. For instance, we still do not understand how distinct tumors are able to differentially modulate the unique commensal microbial species and *vice versa*. Furthermore, whether the first perturbation is caused by the cancer or the microbiome itself is still shrouded in mystery. Bacterial quorum sensing, signaling through extracellular vesicles, circulating metabolites and cytokines could all be involved in providing the initial cues; however, these hypotheses need to be tested in preclinical models to elucidate the mechanisms governing this crosstalk. There is also a need for further integration of viral and fungal species in cancer research to figure out the exact nature of inter-commensal dynamics and their individual as well as combined roles in tumor progression and immunomodulation. Recent studies have made some headway in resolving the question of local *vs* distant microbial immunomodulation (26, 52), suggesting discrete actions of adaptive immune cells in the mucosal and systemic compartments. Hence, it is likely that distant effects are being mediated through microbial products like metabolites and cytokines, but more rigorous studies are required to prove this hypothesis.

The existence of microbial epitopes with homology to tumor associated antigens had an uncertain stature in the field of ‘oncomicrobiotics’ till only a few years ago, but has now been shown to have a definitive role in anti-tumor immunity, as a result of seminal work from the team of Dr. Zitvogel (118). Their observations regarding the presence of such epitopes across multiple cancer types open the door towards widespread characterization of commensal proteins to identify possible antigens which can lead to the generation of tumor-specific microbial vaccines.

Stromal elements sculpt the tumor immune environment through paracrine cues involving cytokines and metabolic signals. CAFs in the stroma have been implicated as the main source of tumor resistance to anti-cancer immune response, and therapies targeting stromal markers are being investigated in clinical trials to synergize with current immunotherapies (228–230). Interestingly, TLR4 signaling in intestinal CAFs can promote tumorigenesis (231). HSCs also highly express TLRs and experience activation under the effects of direct interaction with the gut microbiome or their metabolites (232, 233), contributing directly to hepatic oncogenesis (149, 159). In view of such literature, it is surprising that currently there is no evidence implicating the interaction of microbiome with CAFs in the tumor microenvironment, an area which will surely be explored as we dissect the role of microbiome in tumor development.

Recently, clonally expanded, class switched memory B cells were found to be enriched in tertiary lymphoid structures (TLSs) inside tumors from immunotherapy responder as compared to non-responder melanoma patients. Immunotherapy responders showed distinct B cell gene expression with enhanced anti-tumor immune signaling in the form of CXCR4 signaling, cytokine receptor interaction and chemokine signaling pathways (234, 235). Considering that the B cell repertoire and specificity can be shaped by the microbiome (77) and the microbiome itself can determine the efficacy of cancer immunotherapy, the microbiome–B cell interaction can be further explored in the context of cancer immunotherapy as well.

We have already seen that the microbiome is an important player in epigenetic regulation of cancer-driving mutations (5, 236). The relationship of cancer metagenomics with microbial composition should also be explored further to delineate the impact of these commensals on molecular subtypes of different cancers (237).

Functional annotation of sequenced microbial taxa is a challenging prospect as members of the same taxa might have distinct phenotypes under different physiological conditions and in the setting of different tumors. For example, SCFA production through gut microbes has been shown to exert an immunosuppressive effect in the colon in IBD patients (238) while it also seems to be associated with improved immunotherapy efficacy in cancer patients (150). With the advent of a multiomics approach even in the field of microbiome research, integration of transcriptomics, proteomics and metabolomics to the metagenomic sequencing should be pursued to comprehensively characterize the host–microbiome interactome and identify unique functional subsets of the candidate microbes.

The most exciting aspect of elucidation of this microbiome-cancer axis is the tantalizing translational possibilities. The gut microbiome is an easily accessible biomarker and is amenable to swift manipulations using existing therapies like antibiotics and probiotics. Moreover, it can confer a unique individual specific signature, which opens up the realm of personalized and precision medicine. Leveraging these advantages, researchers are pursuing novel breakthroughs in microbiome-based diagnostics and therapeutics. Identification of unique tumor-specific microbial and/or metabolomic signatures along with co-existent immune phenotypes can be utilized to generate models for early screening and risk stratification in cancer patients (239–243). Leveraging the TCGA database for whole genome and whole transcriptome sequencing information on 18,116 samples from 33 cancer types, Poore et al. were able to detect microbial signatures in the sequencing samples of both tissue and blood. Using machine learning, the authors generated a highly discriminatory tool to separate patients with cancer from healthy volunteers. The microbiome-based signature was able to identify tumor samples without any known oncogenic mutation, underscoring the exciting potential of microbiome based diagnostic tools in cancer screening and diagnosis (10).

The ability of the microbes to home towards tumor cells and interact with the immune cells is a potential source of microbial-vaccines and targeted therapies. Chowdhury et al. (244) recently engineered non-pathogenic strains of *E coli* which could release anti-tumor immunotherapeutic agents in the form of anti-CD47 immunotherapy on spontaneous quorum-lysis in the TME. This effect was present not only on intra-tumoral injections, but also on intravenous administration, with bacteria exclusively colonizing the tumors and not organs like liver, kidney, and spleen. The authors also noted that there was a stimulation of the systemic immune response along with the anti-tumor response in the TME, with decreased metastasis burden in the lungs as well (244). Another interesting aspect of this therapeutic strategy is the adjuvant stimulation of the immune system through bacterial products disseminated in the microenvironment along with the targeted immunotherapy being delivered, highlighting the multi-faceted potential of such approaches in tumor therapy (244). Similarly, it might be possible to use re-engineered commensals expressing the desired phenotype to specifically modulate the microbiome and combat disease progression.

The microbiome is shaped by various factors including diet, age, health and disease, stress and lifestyle factors. Major oncogenic risk factors like obesity (6), smoking (7), high fat diet (8) and alcohol consumption (9) have now been shown to cause significant microbial dysbiosis. Even more intriguing is the finding that gut microbial dysbiosis can mediate the tumor promoting effects of risk factors like high fat diet (245) and smoking (246). These findings raise the question whether microbiome modulation strategies can be explored as primary prevention measures against certain cancers, a question which needs to be answered through epidemiological and prospective studies.

Spatio-temporal distribution of B and T cell subsets is significantly altered as precursor lesions transition to invasive carcinoma and during progression of tumor stages (247, 248). Microbial populations also change dynamically during transition from precursor lesions to invasive lesions (249). In the context of tumor-initiating effects of the microbiome, evaluating its effects on temporal shifts in immune composition of TME will be important for gaining insights into progression of pre-invasive lesions into invasive carcinomas. Similar to cancer initiation, there are reports of association of microbial species with metastatic and recurrent tumors. *F. nucleatum* has been shown to promote CRC mets *via* upregulation of autophagy signaling (250). On the other hand, probiotic administration was able to reduce recurrence in a RCT of superficial bladder cancer patients (251). The role of gut microbiome in cancer recurrence and metastasis needs to be further investigated in epidemiological studies as well as through use of immunocompetent pre-clinical models (252, 253). As technological breakthroughs make high throughput sequencing more efficient and accessible, we have started generating vast amounts of metadata through metagenomic, metabolomics, and even transcriptomic sequencing of the resident flora. Curating integrated databases from human and murine studies and applying computational bioinformatics will enable us to create a comprehensive reference database containing phylogenetic and functional alterations of the microbiome along with disease stages and host-specific cellular responses, serving as a fountainhead which can drive the next phase of cancer–microbiome research (254).

## Conclusion

The microbiome is inextricably integrated into the cancer-immune crosstalk. The microbes interact with the host and the tumor in a diverse and context-specific manner, conferring individual and tumor-specificities to the immunomodulatory phenotype observed. Elucidation of the unique mechanisms through which these manipulations occur can help devise targeted and personalized tools for cancer diagnosis, therapy and prevention. The era of immuno-onco-microbiotics is well and truly underway.

## Author Contributions

TJ and PS were involved in the initial literature review for the manuscript. TJ, PS, and VD contributed to writing of the manuscript. TJ, PS, AA, SV, and VD all were responsible for editing the manuscript. SV and VD provided critical review of the contents of the manuscript. All authors contributed to the article and approved the submitted version.

## Funding

This work is supported by NIH grant R01 DK 111834 and DOD grants W81XWH-17-1-0392 and W81XWH-16-1-0570.

## Conflict of Interest

The authors declare that the research was conducted in the absence of any commercial or financial relationships that could be construed as a potential conflict of interest.
